# Child maltreatment and cardiovascular disease: quantifying mediation pathways using UK Biobank

**DOI:** 10.1186/s12916-020-01603-z

**Published:** 2020-06-12

**Authors:** Frederick K. Ho, Carlos Celis-Morales, Stuart R. Gray, Fanny Petermann-Rocha, Donald Lyall, Daniel Mackay, Naveed Sattar, Helen Minnis, Jill P. Pell

**Affiliations:** 1 Institute of Health and Wellbeing, University of Glasgow, R305 House 1, Public Health, 1 Lilybank Gardens, Glasgow, G12 8RZ UK; 2 Institute of Cardiovascular and Medical Sciences, University of Glasgow, Glasgow, UK; 3 Centre for Exercise Physiology Research (CIFE), Universidad Mayor, Santiago, Chile; 4 Research Group in Education, Physical Activity and Health (GEEAFyS), Universidad Católica del Maule, Talca, Chile

**Keywords:** Child maltreatment, Cardiovascular disease, Depression, Mediation

## Abstract

**Background:**

Child maltreatment is associated with cardiovascular disease (CVD), but mediation pathways have not been fully elucidated. The aim of the current study was to determine and quantify the underlying pathways linking child maltreatment and CVD.

**Methods:**

We conducted a retrospective cohort study using the UK Biobank. The number and types of child maltreatment, including abuse and neglect, were recalled by the participants. Lifestyle, biological, physical, and mental health factors measured at baseline were explored as potential mediators. Incident CVD was ascertained through record linkage after baseline measurement. Age, sex, ethnicity, area-based deprivation, and education level were adjusted for as confounders. Cox proportional hazard models were conducted to test for associations between child maltreatment and incident CVD.

**Results:**

A total of 152,040 participants who completed the child maltreatment assessment were included in the analyses, and one third reported at least one type of child maltreatment. There was a dose-response relationship between the number of maltreatment types and incident CVD. On average, each additional type of child maltreatment was associated with an 11% (95% CI 8–14%, *P* < 0.0001) increased risk of CVD. The majority (56.2%) of the association was mediated through depressive symptoms, followed by smoking (14.7%), high-density lipoprotein cholesterol (8.7%), and sleep duration (2.4%).

**Conclusion:**

Child maltreatment is associated with incident CVD through a combination of mental health, lifestyle, and biological pathways. Therefore, in addition to interventions to reduce the occurrence of child maltreatment, attention should be targeted at promoting healthy lifestyles and preventing, identifying, and treating depression among children and adults who have previously been maltreated.

## Introduction

Cardiovascular disease (CVD) is multifactorial in its aetiology, with a broad range of risk factors. The more proximal risk factors include dyslipidaemia, hypertension, and diabetes which are often related to lifestyle behaviours such as smoking and physical activity [1]. More recently, evidence has shown that people exposed to traumatic experiences were more likely to develop CVD [2, 3]. Traumatic experiences include child maltreatment, which covers all types of neglect and physical, emotional, and sexual abuse that occur in childhood and results in adverse health and well-being. Child maltreatment is an important component of adverse childhood experiences (ACEs) [4].

Emerging evidence has shown associations between child maltreatment and cardiovascular risk factors and cardiovascular disease. For example, a cross-sectional study has shown a dose-response relationship between child maltreatment and myocardial infarction, a common presentation of CVD [5], and a retrospective study reported an association of child maltreatment with self-report of physician-diagnosed ischaemic heart disease [6].

Indeed, the American Heart Association has published a scientific statement recognising the importance of child maltreatment in cardiometabolic outcomes as well as proposing a conceptual model between child maltreatment and CVD [7]. The hypothesised pathways suggested child maltreatment may affect health behaviours (such as physical activity and smoking), physiologic factors, and mental health which, in turn, influence proximal cardiovascular risk factors (such as obesity and diabetes) and finally the onset of CVD. These pathways were supported by a review [8] and several studies, for example, on the association of child maltreatment with smoking [9], depression [10], and cardiovascular risk factors [11]. In fact, the Nurses’ Health Study 2 has shown adult health-related factors, such as body mass index, smoking, alcohol use, and depression, accounted for 79% and 63% of the association of severe physical and sexual abuse with CVD, respectively [12]. However, very few studies have examined the mediators simultaneously and compared the relative importance between them.

Understanding the extent to which the association between child maltreatment and CVD is mediated by different pathways could inform the development and targeting of interventions to protect the long-term health of affected individuals. The aim of the current study was, therefore, to use data on UK Biobank participants to study whether, and to what extent, different pathways mediated the association between child maltreatment and CVD.

## Methods

### Study design and participants

Between 2007 and 2010, the UK Biobank recruited 502,506 participants from the general population. Participants attended one of 22 assessment centres across England, Scotland, and Wales where they completed a self-administered, touch-screen questionnaire and a face-to-face interview, and trained staff took a series of measurements including height, weight, and blood pressure. Ethnicity, education level, sleep duration, television viewing time, smoking status, and alcohol intake were self-reported. Townsend area deprivation index was derived from the postcode of residence using aggregated data on unemployment, car and home ownership, and household overcrowding [13]. Blood pressure was measured by a trained nurse. Physical activity was self-reported using the validated International Physical Activity Questionnaire [14]. Grip strength was measured to the nearest 0.1 kg using a Jamar J00105 hydraulic hand dynamometer and the mean value from both hands used in the analyses. Height was measured to the nearest centimetre, using a Seca 202 stadiometer, and body weight to the nearest 0.1 kg, using a Tanita BC-418 body composition analyser. Body mass index (BMI) was calculated as weight/height^2^, and the World Health Organization’s criteria were used to classify BMI into underweight (< 18.5 kg/m^2^), normal weight (18.5 to 24.9 kg/m^2^), overweight (25 to 29.9 kg/m^2^), and obese (≥ 30.0 kg/m^2^). Abdominal obesity was defined as waist-hip ratio > 0.85 for women and > 0.90 for men. Biomarkers were performed at a dedicated central laboratory between 2014 and 2017. In this study, we have selected high-density lipoprotein [HDL] cholesterol, low-density lipoprotein [LDL] cholesterol, glycated haemoglobin, cystatin C, and gamma-glutamyltransferase as potential mediators. HDL and LDL are cholesterols related to CVD and lifestyle factors such as smoking and obesity; glycated haemoglobin is a marker for diabetes and related to obesity; cystatin C is a marker of kidney function and related to diet, smoking, and body weight; gamma-glutamyltransferase is a marker of liver function and related to alcohol drinking. Details of these measures and assay performances can be found online in the UK Biobank showcase and protocol [15].

The participants were subsequently invited to complete a web-based questionnaire [16] on mental health-related items: child maltreatment using the Childhood Trauma Screener, depressive symptoms using the Patient Health Questionnaire 9-items (PHQ-9) [17], anxiety symptoms using the Generalised Anxiety Disorder 7-items (GAD-7) [18], and other mental health problems using the Composite International Diagnostic Interview–Short Form [19]. Approximately one third (31.31%, *n* = 157,348) of the participants completed the online follow-up. These participants were generally younger, more likely to be female and White, and had healthier lifestyles (Additional file 1: Table S1). Of these, 3714 (2.36%) who did not complete all of the child maltreatment items and 1594 (1.01%) who had missing sociodemographic data were excluded from the analyses. Therefore, this study comprised 152,040 participants.

### Child maltreatment

Child maltreatment was assessed using the Childhood Trauma Screener (CTS) [20], a shortened version of the Childhood Trauma Questionnaire (CTQ) [21]. It consists of one 5-point Likert scale item for each of five types of child maltreatment: physical abuse, physical neglect, emotional abuse, emotional neglect, and sexual abuse, and has been validated against the CTQ with good overall (*r* = 0.88) and satisfactory type-specific correlations (*r* = 0.55–0.87) [22]. The CTQ is a widely used instrument for measuring child maltreatment and has been validated against the actual record of abuse and neglect [20, 23]. The threshold values on the Likert scale derived from the validation study [20] were used to define the presence or absence of each type of child maltreatment (Additional file 1: Table S2). In this study, the primary exposure variable was the number of types of child maltreatment (range 0 to 5) as it reflects the dimensions of maltreatment.

### Outcome ascertainment

Outcomes were ascertained through individual-level record linkage of the UK Biobank cohort. Date and cause of death were obtained from death certificates held by the National Health Service Information Centre (England and Wales) and the National Health Service Central Register Scotland (Scotland). Date and cause of hospital admissions were obtained through record linkage to Health Episode Statistics (England and Wales) and Scottish Morbidity Records (Scotland). Detailed information about the linkage procedures can be found at http://content.digital.nhs.uk/services. At the time of analysis, hospital admission data were available up to 31 March 2017. We defined incident CVD as death or hospital admission for ischaemic heart disease (including myocardial infarction), atrial fibrillation, heart failure, or stroke (ICD-10 [international classification of diseases, 10th revision] codes I20, I21, I25, I48, I50, I60, I61, I63, I64) after baseline assessment. These outcomes were chosen as they comprised the majority of the CVD events.

### Statistical analyses

Cox proportional hazard models were used to analyse the association between child maltreatment and incident CVD, with the results reported as hazard ratios (HRs) and 95% confidence intervals (CIs). The outcome variable comprised both the event status and time-to-event. The models were adjusted for age at baseline assessment, sex, ethnicity, deprivation index, and education level as potential confounders. Subgroup analyses were conducted for sociodemographic factors: sex, age group (38–50, 51–60, and 61–72 years), education level (with vs. without university degree), and area-based deprivation index (≥ vs. < median). We selected these factors because age could affect the reporting of maltreatment, sex could affect the type of maltreatment, and socioeconomic status might affect the ability to cope with these maltreatment experiences.

We studied four groups of potential mediators: lifestyle (sleep duration [categorical variable]; any smoking and alcohol drinking > 14 units a week [binary variables]; physical activity and television viewing [continuous variables]), physical measurements (BMI categories [categorical variable], abdominal obesity [binary variable], and systolic blood pressure and grip strength [continuous variables]), mental health (diagnosed depression, anxiety, and schizophrenia [binary variables]; depressive and anxiety symptoms [continuous variables]; any psychotic experience, behavioural addiction, drug addiction, or self-harm behaviours [binary variables]), and biomarkers (HDL cholesterol, LDL cholesterol, glycated haemoglobin, cystatin C, and gamma-glutamyltransferase [continuous variables]). Sleep duration and alcohol drinking variables were categorised based on the UK recommendations because of their potential nonlinear association with CVD. All potential mediators were selected because of their association with child maltreatment and/or CVD. These groups of mediators were adjusted in the Cox models to examine whether, and to what extent, the HRs between child maltreatment and CVD were attenuated. In addition, the model-based mediation analysis framework by Tingley et al. was also conducted [24]. Firstly, CVD incidence was regressed by the primary exposure variable (number of child maltreatment), all potential mediators, and sociodemographic factors in a Weibull regression model with a robust standard error. Weibull regression was chosen for mediation analysis because of its superior statistical properties over proportional hazard models [25]. Only the potential mediators reaching statistical significance (*α* = 0.05) were further investigated. The filtered potential mediators were then regressed by child maltreatment and other covariates (mediator model) in either logistic (for binary mediators) or multiple linear (for other mediators) models. The results of the outcome and mediator models were then combined to estimate the proportion of mediation. Potential mediators were mutually adjusted to avoid overestimation of mediating effects. Quasi-Bayesian estimation with 1000 iterations was used for estimating the *P* values of the mediating effect.

Four sensitivity analyses were conducted. Firstly, the standardised CTS score was used in a penalised regression spline to examine if its relationship with CVD was consistent with that based on the categorised maltreatment variables. Secondly, the association between maltreatment and CVD by different adjustment models was re-analysed after excluding participants who had prevalent CVD at baseline assessment (*n* = 6684). Thirdly, in the mediation analysis, because biomarkers can be dependent on sex, the mediation analysis was replicated with sex-specific *z*-scores of biomarkers. Lastly, because biomarkers can lie on the pathways between lifestyle and CVD, the mediation analysis was replicated without them. This helped to identify lifestyle factors that might be masked by adjustment of biomarkers. Proportional hazard assumptions were verified by statistical tests based on Schoenfeld residuals. Distributional assumption of Weibull regression was examined using the Kaplan-Meier estimate of the residuals. Missing data was handled using complete case analysis. All analyses were conducted using R version 3.5.3 with packages *survival* and *mediation*.

## Results

This study included 152,040 (mean [SD] age 55.92 [7.73] years; 56.34% female) UK Biobank participants. One third (*n* = 50,602) of the participants reported at least one type of child maltreatment, and 12.95% reported multiple types. Participants who reported multiple child maltreatment were generally younger; more deprived; more physically active; spent more time watching television; were more likely to be female, smokers, and obese; and have short (< 6 h) or long (> 9 h) sleep duration (Table 1). They also had lower systolic blood pressure, grip strength, and HDL cholesterol, and reported more depressive and anxiety symptoms.

**Table 1:** Participant characteristics

	Number of child maltreatment types			
	None (*n* = 101,438)	One (*n* = 30,915)	Two (*n* = 11,625)	Three or more (*n* = 8062)
Mean (SD) age, years	56.16 (7.70)	55.81 (7.73)	55.25 (7.80)	54.17 (7.71)
Female	55,938 (55.1)	17,134 (55.4)	7082 (60.9)	5509 (68.3)
Ethnicity				
White	99,415 (98.0)	29,843 (96.5)	11,061 (95.1)	7445 (92.3)
Mixed	361 (0.4)	199 (0.6)	101 (0.9)	127 (1.6)
South Asian	656 (0.6)	323 (1.0)	147 (1.3)	125 (1.6)
Black	450 (0.4)	237 (0.8)	155 (1.3)	218 (2.7)
Chinese	144 (0.1)	108 (0.3)	53 (0.5)	43 (0.5)
Others	412 (0.4)	205 (0.7)	108 (0.9)	104 (1.3)
Socioeconomic status				
Mean (SD) deprivation index	− 1.89 (2.73)	− 1.55 (2.89)	− 1.26 (3.03)	− 0.78 (3.25)
Education level				
College or university degree	47,464 (46.8)	13,809 (44.7)	4936 (42.5)	3219 (39.9)
A levels/AS levels or equivalent	13,885 (13.7)	4125 (13.3)	1531 (13.2)	1030 (12.8)
O levels/GCSEs or equivalent	20,041 (19.8)	6114 (19.8)	2353 (20.2)	1642 (20.4)
SEs or equivalent	3445 (3.4)	1224 (4.0)	533 (4.6)	417 (5.2)
NVQ or HND or HNC or equivalent	4848 (4.8)	1669 (5.4)	650 (5.6)	514 (6.4)
Other professional qualifications	5044 (5.0)	1602 (5.2)	596 (5.1)	461 (5.7)
None of the above	6440 (6.3)	2273 (7.4)	968 (8.3)	744 (9.2)
Prefer not to answer	271 (0.3)	99 (0.3)	58 (0.5)	35 (0.4)
Lifestyle factors				
Sleep duration				
< 6 h	913 (0.9)	330 (1.1)	211 (1.8)	161 (2.0)
6–9 h	97,276 (96.1)	29,206 (94.7)	10,730 (92.6)	7211 (89.9)
> 9 h	3075 (3.0)	1304 (4.2)	650 (5.6)	645 (8.0)
Smoking status				
Never	61,136 (60.4)	16,848 (54.6)	5823 (50.2)	3589 (44.7)
Former	33,856 (33.4)	11,524 (37.3)	4671 (40.3)	3397 (42.3)
Current	6279 (6.2)	2488 (8.1)	1101 (9.5)	1049 (13.1)
Alcohol drinking > 14 units/week	40,341 (42.9)	12,215 (42.7)	4263 (40.0)	2635 (35.9)
Mean (SD) MET·min of physical activity/week	2417.23 (2236.73)	2440.37 (2303.14)	2519.91 (2348.86)	2724.99 (2535.82)
Mean (SD) hours of TV viewing/day	2.45 (1.38)	2.48 (1.44)	2.53 (1.50)	2.58 (1.59)
Physical measurements				
BMI categories				
Underweight	569 (0.6)	172 (0.6)	54 (0.5)	53 (0.7)
Normal	39,848 (39.4)	11,521 (37.4)	4105 (35.4)	2600 (32.3)
Overweight	42,157 (41.6)	12,784 (41.5)	4711 (40.6)	3204 (39.8)
Obese	18,663 (18.4)	6358 (20.6)	2734 (23.6)	2184 (27.2)
Abdominal obesity	42,863 (42.3)	13,636 (44.2)	5122 (44.1)	3518 (43.7)
Mean (SD) systolic blood pressure, mmHg	136.70 (18.06)	135.68 (17.97)	134.95 (18.16)	133.65 (18.09)
Mean (SD) grip strength, kg	31.48 (10.70)	31.30 (10.74)	30.37 (10.66)	29.19 (10.38)
Psychiatric/emotional factors				
Depression diagnosed	3683 (3.6)	1919 (6.2)	1013 (8.7)	1016 (12.6)
Anxiety diagnosed	1282 (1.3)	591 (1.9)	279 (2.4)	271 (3.4)
Schizophrenia diagnosed	184 (0.2)	85 (0.3)	58 (0.5)	66 (0.8)
Mean (SD) depressive symptoms (PHQ-9)	2.20 (3.05)	3.23 (3.87)	4.22 (4.65)	5.45 (5.69)
Mean (SD) anxiety symptoms (GAD-7)	1.72 (2.91)	2.49 (3.59)	3.28 (4.18)	4.28 (4.95)
Any psychotic experience	3351 (3.3)	1624 (5.3)	930 (8.2)	1037 (13.4)
Any behavioural addiction	912 (0.9)	596 (2.0)	312 (2.7)	332 (4.2)
Any drug addiction	786 (0.8)	470 (1.5)	283 (2.5)	332 (4.2)
Any self-harm behaviours	2376 (2.3)	1668 (5.4)	1101 (9.5)	1390 (17.4)
Mean (SD) biomarkers				
HDL cholesterol, mmol/L	1.49 (0.38)	1.47 (0.38)	1.47 (0.38)	1.47 (0.38)
LDL cholesterol, mmol/L	3.58 (0.84)	3.57 (0.84)	3.56 (0.84)	3.57 (0.87)
Glycated haemoglobin, mmol/mol	35.26 (5.40)	35.36 (5.70)	35.55 (6.32)	35.57 (6.09)
Cystatin C, mg/L	0.88 (0.15)	0.89 (0.15)	0.88 (0.15)	0.88 (0.17)
Gamma-glutamyltransferase, U/L	33.79 (34.94)	34.33 (35.71)	34.46 (36.36)	34.79 (35.76)

Numbers presented are *n* (%) unless otherwise specified

Of the five types of child maltreatment, emotional neglect (22.11%) was the most commonly reported whilst physical neglect (5.61%) was the least (Additional file 1: Tables S2 and S3). Compared with men, women were more likely to report emotional and sexual abuse and less likely to report physical abuse. Younger participants reported child maltreatment more commonly than older participants across all types except for physical neglect.

**Fig. 1:**
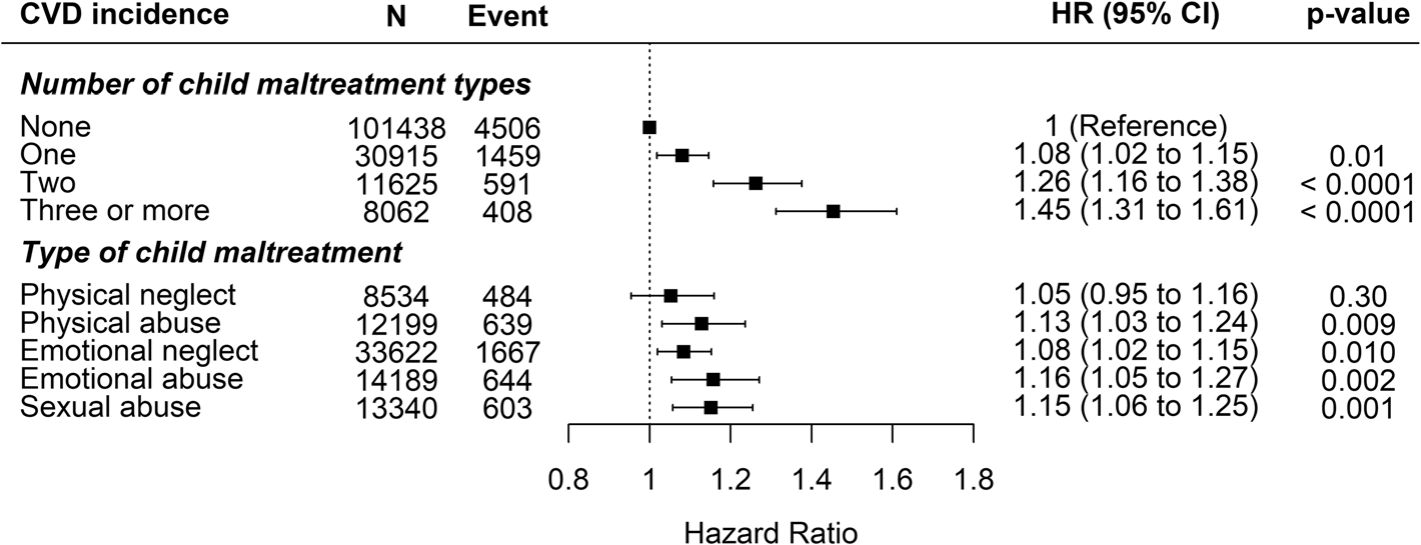
Association of child maltreatment with CVD incidence. Adjusted for age, sex, ethnicity, deprivation index, and education level. Type of child maltreatment was mutually adjusted

After adjusting for sociodemographic characteristics, child maltreatment was significantly associated with incident CVD with evidence of a dose-response relationship (Fig. 1). Compared with participants who did not report maltreatment, those exposed to three or more types were the most likely to develop CVD (HR 1.45 [95% CI 1.31–1.61], *P* < 0.0001), followed by those exposed to two (1.26 [1.16–1.38], *P* < 0.0001) then one (1.08 [1.02–1.15]; *P* = 0.01). On average, each additional type of child maltreatment was associated with an 11% (95% CI 8–14%, *P* < 0.0001) increase in the risk of CVD (Additional file 1: Figure S1), a finding largely consistent among subgroups (Additional file 1: Figure S2). No significant differences between the subgroups were detected (all *P*_Interaction_ > 0.19). The associations with CVD were the strongest for emotional (1.16 [1.05–1.27], *P* = 0.002) and sexual (1.15 [1.06–1.25] *P* = 0.001) abuse (Fig. 1). Of the cardiovascular outcomes, child maltreatment was the most strongly associated with heart failure (1.19 [1.05–1.35] *P* = 0.006) and least strongly with stroke (1.10 [1.02–1.18], *P* = 0.01), although confidence intervals overlapped (Additional file 1: Figure S1). The association between child maltreatment score and CVD showed a linear dose-response relationship when the score was higher than the mean (Additional file 1: Figure S3).

**Fig. 2:**
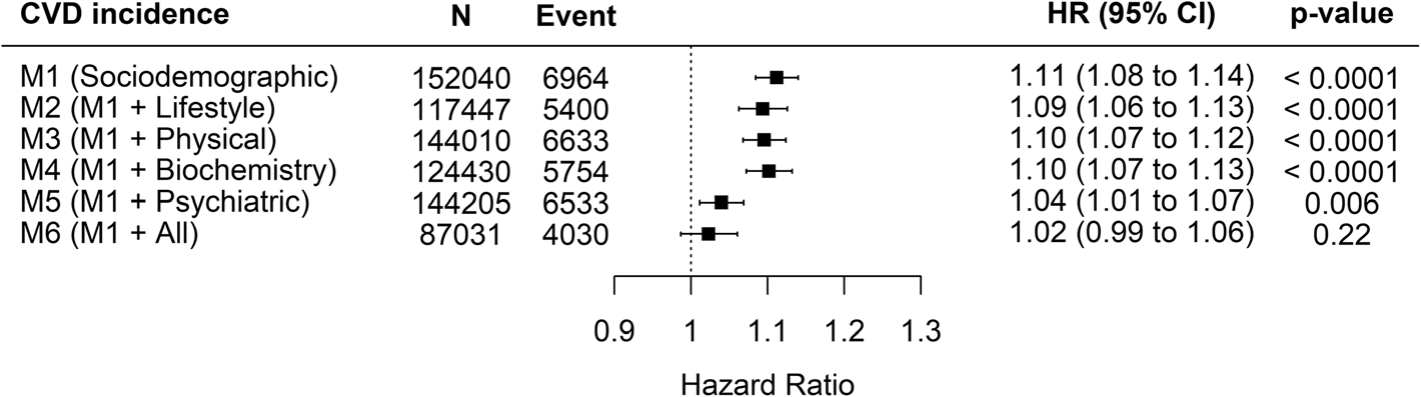
Association of numbers of child maltreatment with CVD by adjustment schemes. M1: adjusted for age, sex, ethnicity, area-based deprivation index, and education level; M2: M1 + lifestyle factors—sleep duration, smoking, alcohol drinking, physical activity, and TV viewing; M3: M1 + physical measurements—BMI categories, abdominal obesity, systolic blood pressure, and hand grip strength; M4: M1 + biomarkers—HDL and LDL cholesterols, glycated haemoglobin, cystatin C, gamma-glutamyltransferase; M5: M1 + psychiatric/emotional factors—diagnoses of depression, anxiety, schizophrenia, depressive symptoms, anxiety symptoms, any psychosis experience, any behavioural addiction, any drug addiction, and any self-harm behaviours; M6: M1 + all potential mediators

Adjustment for the four groups (lifestyle factors, physical measurements, biomarkers, and psychiatric factors) of potential mediators fully accounted for the association between child maltreatment and CVD (before adjustment 1.11 [1.08–1.14], *P* < 0.0001; after adjustment 1.02 [0.99–1.06], *P* = 0.22) (Fig. 2). The strongest attenuation was seen following adjustment for psychiatric/emotional factors (before adjustment 1.11 [1.08–1.14]; after adjustment 1.04 [1.01–1.07], *P* = 0.006). The associations and attenuation of associations were largely consistent when we removed participants with prevalent CVD at baseline assessment (Additional file 1: Figure S4).

**Fig. 3:**
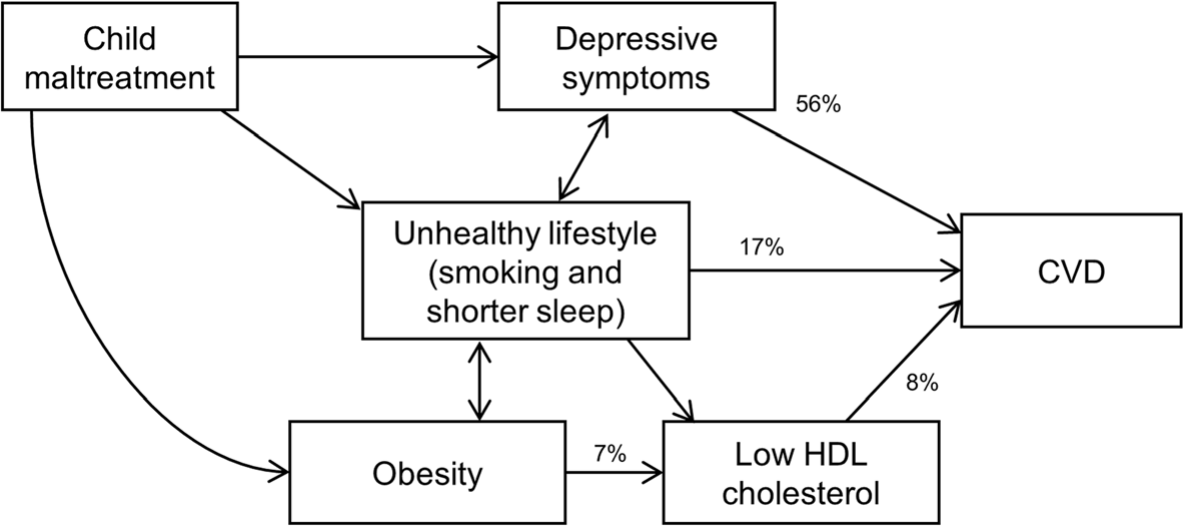
Hypothesised causal pathways between child maltreatment and CVD based on mediation analysis shown in Tables 2 and S6. Direct association from child maltreatment to CVD, and some intercorrelations (e.g. between depressive symptoms and obesity) were omitted for clarity

The outcome model of the mediation analysis is shown in Additional file 1: Table S4. Short sleep duration, smoking status, television watching, obesity, high systolic blood pressure, low grip strength, depressive symptoms, low HDL cholesterol, glycated haemoglobin, cystatin C, and gamma-glutamyltransferase concentrations were significantly associated with a higher risk of CVD after adjusting for child maltreatment, sociodemographic characteristics, and other potential mediators. Television viewing, low grip strength, glycated haemoglobin, cystatin C, and gamma-glutamyltransferase were no longer predicted by child maltreatment after mutual adjustment and were therefore not considered further in the mediation analyses (Table 2). Systolic blood pressure was also dropped from the mediation analysis because it was predicted by fewer types of child maltreatment. The final model included five potential mediators: short sleep duration, smoking status, obesity, depressive symptoms, and HDL cholesterol concentration. Except for obesity, all were found to be significant mediators (*P* < 0.05), with depressive symptoms mediating the majority (56.2%) of the association, followed by smoking status (14.7%), HDL cholesterol concentration (8.7%), and short sleep duration (2.4%) (Table 2). Rerunning the model using sex-specific biomarker *z*-scores yielded similar results (Additional file 1: Table S5). When the mediation analysis was repeated without the biomarkers (including HDL cholesterol concentration), it produced similar findings, except that obesity became a significant mediator, explaining 7.3% of the association between child maltreatment and CVD (Additional file 1: Table S6). The hypothesised direct acyclic diagram based on the mediation analyses is shown in Fig. 3. Tests for proportional hazard assumptions showed no evidence for violation (*P* = 0.08–0.91; global *P* = 0.50).

**Table 2:** Mediation analysis of the relationship between number of child maltreatment and CVD

	Outcome model^a^		Mediator model^b^		Percent mediated	*P*
	HR (95% CI)	*P*	OR/*β* (95% CI)	*P*		
Short sleeper	1.24 (1.10, 1.41)	0.0007	1.14 (1.11, 1.18)	< 0.0001	2.4	0.04
Smoker/ex-smoker	1.13 (1.07, 1.20)	< 0.0001	1.20 (1.18, 1.21)	< 0.0001	14.7	0.02
Obesity	1.15 (1.08, 1.22)	< 0.0001	1.04 (1.03, 1.06)	< 0.0001	2.0	0.07
TV viewing time	1.03 (1.00, 1.06)	0.03	0.00 (− 0.01, 0.00)	0.13	–	–
Systolic blood pressure	1.12 (1.09, 1.15)	< 0.0001	− 0.02 (− 0.02, − 0.01)	< 0.0001	–	–
Handgrip strength	0.96 (0.93, 1.00)	0.08	0.00 (− 0.01, 0.00)	0.69	–	–
Depressive symptom	1.18 (1.16, 1.21)	< 0.0001	0.22 (0.22, 0.23)	< 0.0001	56.2	< 0.0001
HDL cholesterol	0.85 (0.82, 0.88)	< 0.0001	− 0.02 (− 0.02, − 0.01)	< 0.0001	8.7	0.03
Glycated haemoglobin	1.10 (1.08, 1.12)	< 0.0001	0.00 (0.00, 0.01)	0.31	–	–
Cystatin C	1.09 (1.07, 1.11)	< 0.0001	0.00 (0.00, 0.01)	0.10	–	–
Gamma-glutamyltransferase	1.05 (1.03, 1.07)	< 0.0001	0.00 (0.00, 0.01)	0.28	–	–

ORs were presented for short sleeper, smoker/ex-smoker, and obesity

^a^Outcome model: CVD regressed by potential mediators

^b^Mediator model: potential mediator regressed by child maltreatment

## Discussion

Our study demonstrated a significant association between child maltreatment and incident CVD with evidence of a dose-response relationship. Depressive symptoms, smoking, HDL cholesterol concentration (probably mostly via upstream obesity), and short sleep duration mediated 80.2% of the association. Our findings suggest that child maltreatment may predispose people to poor mental health and an unhealthy lifestyle which, in turn, predispose to CVD.

### Comparison with existing literature

Previous prospective cohort studies of the association between child maltreatment, or ACEs more broadly, and CVD have been obliged to use intermediate cardiometabolic risk markers, rather than an incident disease, as the endpoints due to insufficiently long follow-up [11]. A number of retrospective cohort studies have shown an association with CVD but have relied on self-reported endpoints [6]. Our findings are consistent with a retrospective cohort study which reported that traditional risk factors (smoking, physical inactivity, BMI, diabetes, and hypertension), as a group, attenuated the association between ACEs and self-reported IHD by 19% and psychological factors (anger and depressed affect) by 38% [6], and the Nurses’ Health Study 2 which found adult health-related factors (BMI, smoking, alcohol use, depression, etc.) collectively accounted 79% and 63% of the association of severe physical and sexual abuse, respectively [12]. This cohort study extends the existing evidence by analysing a wide range of cardiovascular disease endpoints, including heart failure, an increasingly important public health problem [26].

### Causal pathways

Reviews [15, 27] have suggested that ACEs may increase the risk of CVD through behavioural, emotional, biological, social, and cognitive pathways. Two previous studies have shown that the association between ACEs and CVD was attenuated following adjustment for clusters of behavioural, psychological, and medical factors, but did not study the contribution of individual factors within these clusters [6]. Our study looked at these factors individually and demonstrated that the association was mediated primarily through depressive symptoms. Our findings also suggested that, after adjusting for current symptoms of depression, historical diagnosis of depression was no longer associated with CVD (Additional file 1: Table S4). This could be because the symptom score can capture the severity of depression, or because diagnosed depression is often treated with selective serotonin reuptake inhibitors, a group of medications that was shown to reduce CVD risk in a randomised controlled trial [4].

Because the majority of assessments of the UK Biobank cohort were conducted at baseline, it was not feasible to conduct robust sequential mediation analysis. However, mediators can be causally related to each other. For example, lifestyle/behavioural factors, such as smoking and obesity, may affect serum CVD risk markers, which in turn increases the risk of CVD [28, 29]. We attempted to elucidate additional mediators that were masked by the adjustment of biomarkers through a sensitivity analysis. This revealed obesity to be a mediator of the association between child maltreatment and CVD once HDL cholesterol was removed from the model. Given the close relationship between obesity and HDL cholesterol [30], we hypothesise that children who have been maltreated are at higher risk of becoming obese which, in turn, lowers the HDL cholesterol level and increases the risk of CVD. However, we should note that child maltreatment might be a causative factor in the development of obesity or could be acting through other confounding factors, such as ADHD. ADHD was found to predispose children to being maltreated [31] and to obesity and premature mortality. Notably, we hypothesise that HDL cholesterol may not necessarily be a causal risk factor but more of a risk marker, on the basis that recent trials and genetic studies go against HDL cholesterol being causally linked to CVD per se [32].

### Limitations

As with any observational study, residual confounding, such as genetic traits [33] and ADHD [34], may have occurred. Child maltreatment was recalled by participants rather than captured prospectively, which is a common limitation of studies of childhood experiences with long duration of follow-up. Another common limitation was that information on child maltreatment was limited to type, with no information on severity, even though we identified similar findings when we used the child maltreatment score as the exposure variable. Mediation analysis assumes causality, but it is possible that some mediators, such as depression, influence whether maltreatment is reported since the report of child maltreatment and the assessment of the mediators occurred at the same time [35]. Even though the mediation analysis has considered a wide range of factors, there are still important mediators unexamined, such as post-traumatic stress disorder (PTSD). PTSD was found to have significant overlap with other psychopathologies that we included (such as depression) [36], and therefore, omitting it should not significantly alter the conclusion in this study. We should also note that different types of child maltreatment (for example abuse vs. neglect) may influence health and well-being in distinct ways [37]. Lastly, whilst the UK Biobank cohort is representative of the general population in terms of sociodemographic characteristics, it is not representative in terms of lifestyle [38, 39]. Therefore, whilst effect sizes should be generalisable, summary statistics and estimates of absolute risk should not be generalised.

## Conclusions

Whilst the ultimate aim should be to prevent child maltreatment, individuals who were maltreated in childhood require interventions to protect their long-term health and well-being. Previous studies have demonstrated that adults with a history of child maltreatment are at increased risk of CVD [40], but identification of the main causal pathways is essential to reducing this risk. Our study demonstrates the importance of assessing and helping to improve the mental health and lifestyle of children and adults with previous maltreatment to protect their physical, as well as psychological, health and specifically to reduce their risk of CVD.

## Supplementary information

**Additional file 1: Figure S1.** Association of numbers of child maltreatment with types of CVD. **Figure S2.** Association of number of child maltreatment and incident CVD by subgroups. **Figure S3.** Association of standardised child maltreatment (CTS) score and incident CVD. **Figure S4.** Association of number of child maltreatment and incident CVD after excluding participants with prevalent CVD at baseline assessment. **Table S1.** Characteristics of UK Biobank participants by inclusion in this study. **Table S2.** Participants’ responses to the Childhood Trauma Screener – 5 items (CTS-5). **Table S3.** Prevalence of child maltreatment in UK Biobank. **Table S4.** Association of potential mediators and CVD. **Table S5.** Mediation analysis of number of child maltreatment and CVD using sex-specific biomarker z-scores. **Table S6.** Mediation analysis of number of child maltreatment and CVD excluding biomarkers.

## Data Availability

The data that support the findings of this study are available from the UK Biobank, but restrictions apply to the availability of these data, which were used under licence for the current study, and so are not publicly available. Data are however available from the authors upon reasonable request and with permission from the UK Biobank.
