# Supplementary material for: Child maltreatment and cardiovascular disease: quantifying mediation pathways using UK Biobank
Source: BMC Med. 2020 Jun 12;18:143. doi: 10.1186/s12916-020-01603-z (PMC7291652; doi:10.1186/s12916-020-01603-z)
Supplement: Supplementary file 1 — Additional file 1: Figure S1. Association of numbers of child maltreatment with types of CVD. Figure S2. Association of number of child maltreatment and incident CVD by subgroups. Figure S3. Association of standardised child maltreatment (CTS) score and incident CVD. Figure S4. Association of number of child maltreatment and incident CVD after excluding participants with prevalent CVD at baseline assessment. Table S1. Characteristics of UK Biobank participants by inclusion in this study. Table S2. Participants’ responses to the Childhood Trauma Screener – 5 items (CTS-5). Table S3. Prevalence of child maltreatment in UK Biobank. Table S4. Association of potential mediators and CVD. Table S5. Mediation analysis of number of child maltreatment and CVD using sex-specific biomarker z-scores. Table S6. Mediation analysis of number of child maltreatment and CVD excluding biomarkers. [file 12916_2020_1603_MOESM1_ESM.docx]

Child maltreatment and cardiovascular disease: quantifying mediation pathways using UK Biobank

Frederick K Ho^1*^, Carlos Celis-Morales^1,2*^, Stuart R Gray^2^, Fanny Petermann-Rocha^1,2^, Donald Lyall^1^, Daniel Mackay^1^, Naveed Sattar^2^, Helen Minnis^1^, Jill P Pell^1^

1. Institute of Health and Wellbeing, University of Glasgow, Glasgow, United Kingdom
2. Institute of Cardiovascular and Medical Sciences, University of Glasgow, Glasgow, United Kingdom

Corresponding author: Prof Jill P Pell, R305 House 1, Public Health, 1 Lilybank Gardens, Glasgow G12 8RZ, United Kingdom. Email: [Jill.Pell@glasgow.ac.uk](mailto:Jill.Pell@glasgow.ac.uk). Tel: +441413303239.

^*^ Co-first authors

Figure S1. Association of numbers of child maltreatment with types of CVD


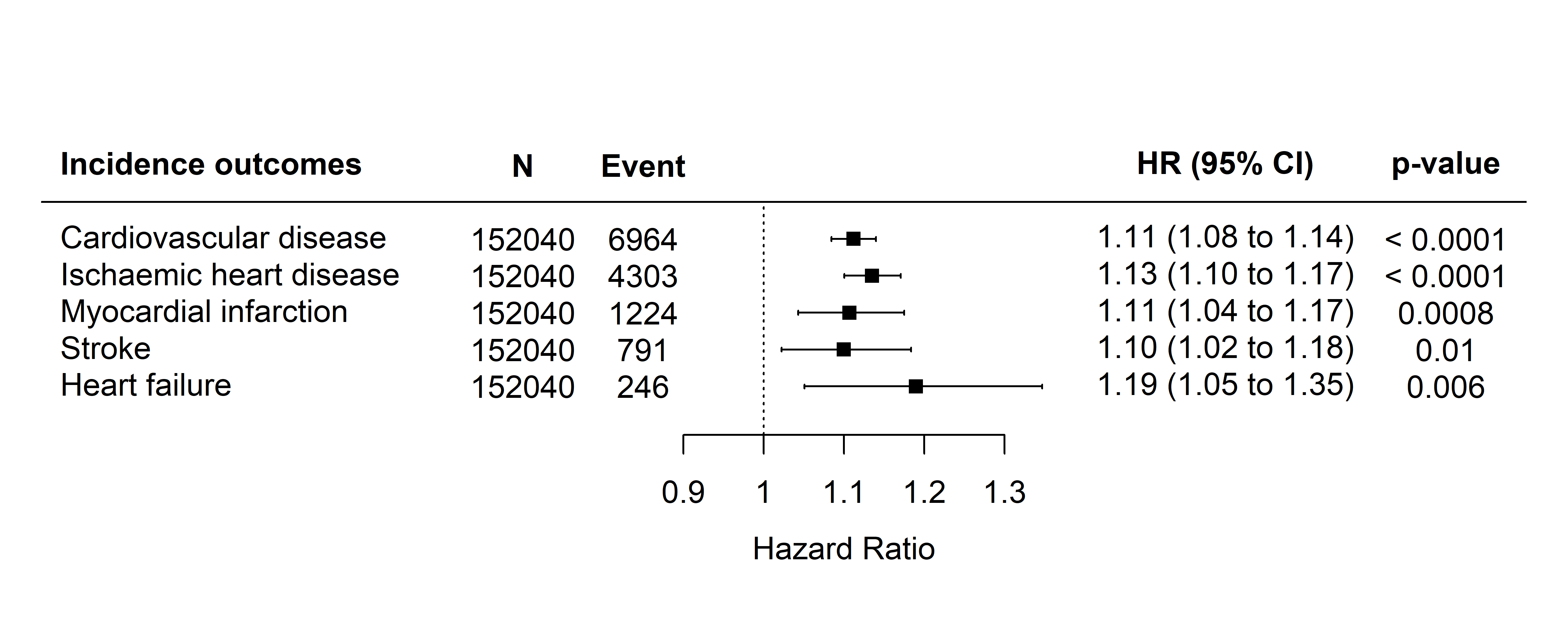


Adjusted for age, sex, ethnicity, area-based deprivation index, and education level.

Figure S2. Association of number of child maltreatment and incident CVD by subgroups


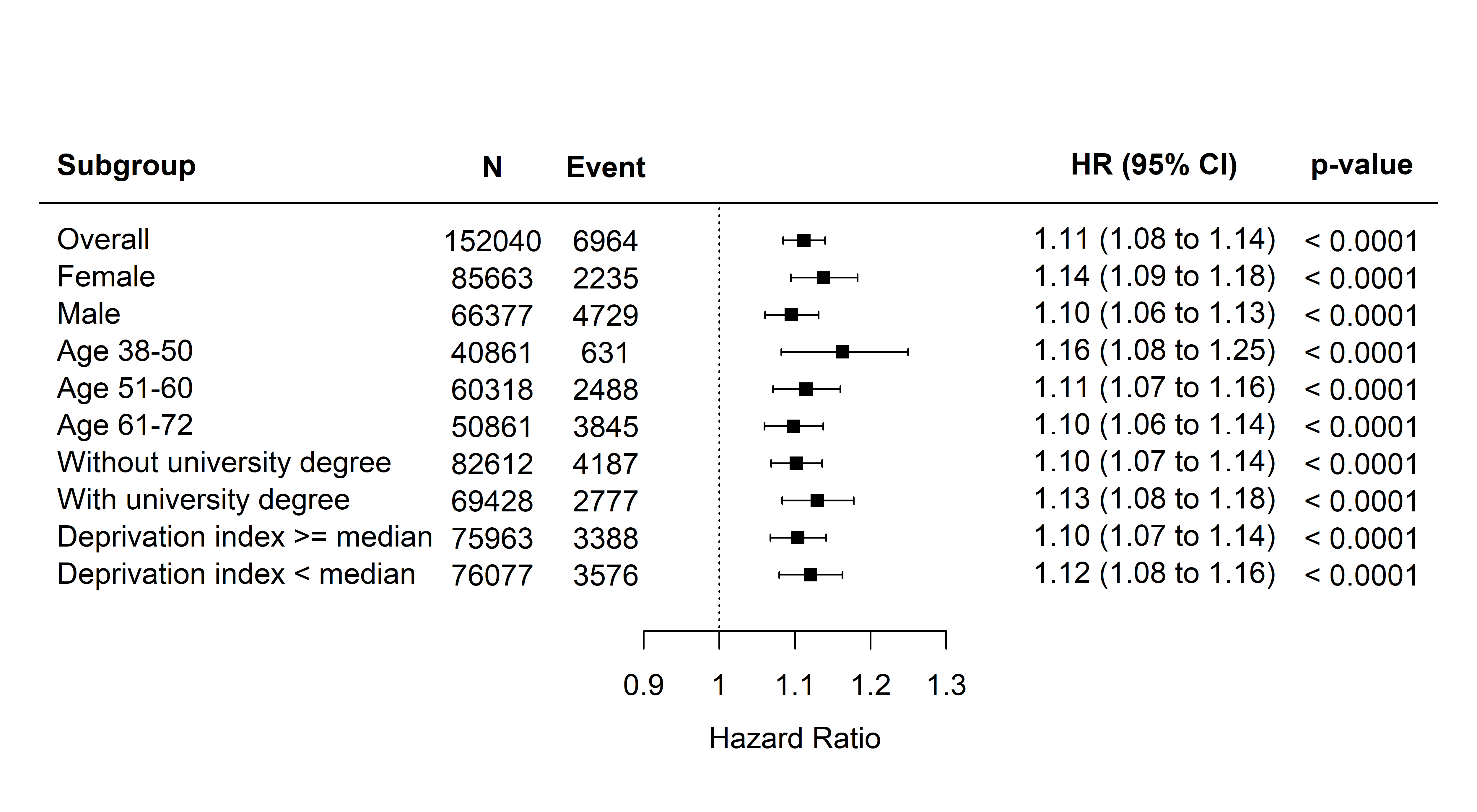


Adjusted for age, sex, ethnicity, area-based deprivation index, and education level

Figure S3. Association of standardised child maltreatment (CTS) score and incident CVD


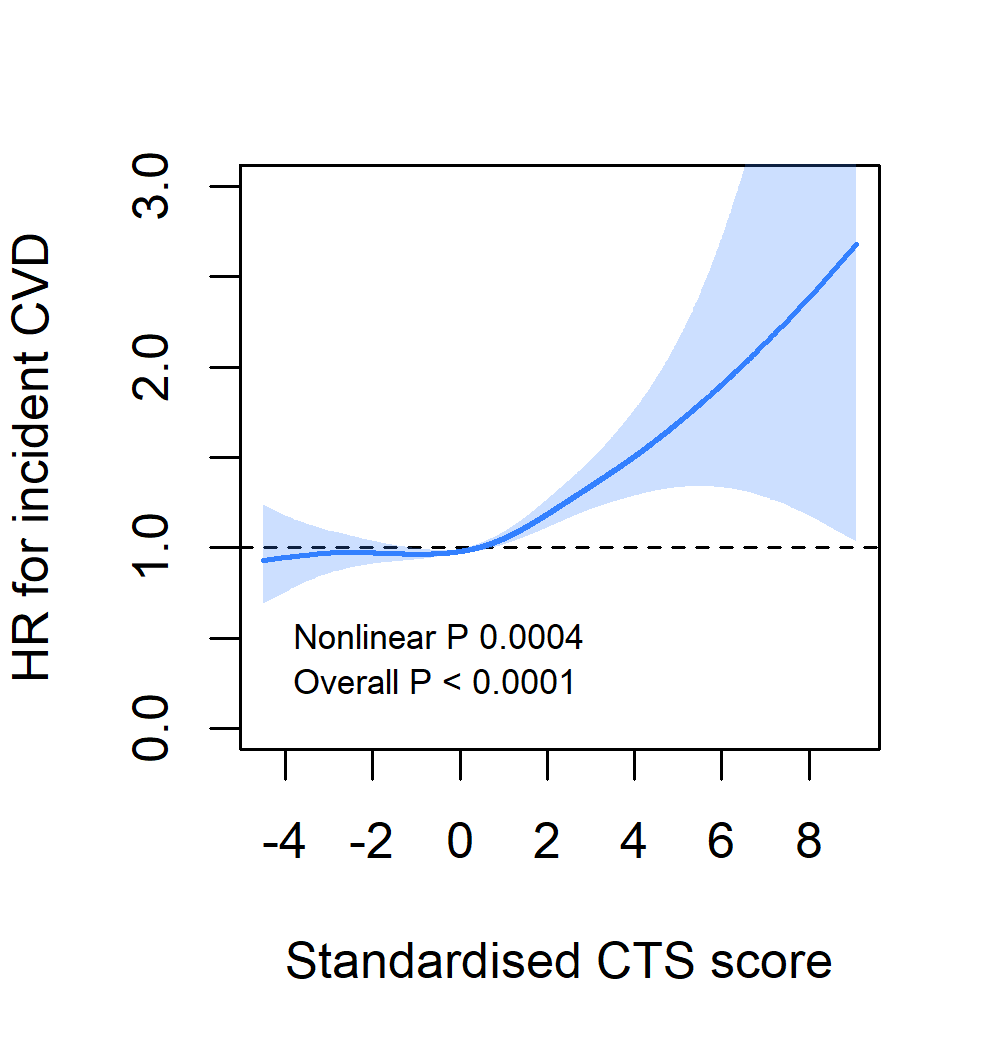


Adjusted for age, sex, ethnicity, area-based deprivation index, and education level. CTS score were standardised to mean and SD.

Figure S4. Association of number of child maltreatment and incident CVD after excluding participants with prevalent CVD at baseline assessment


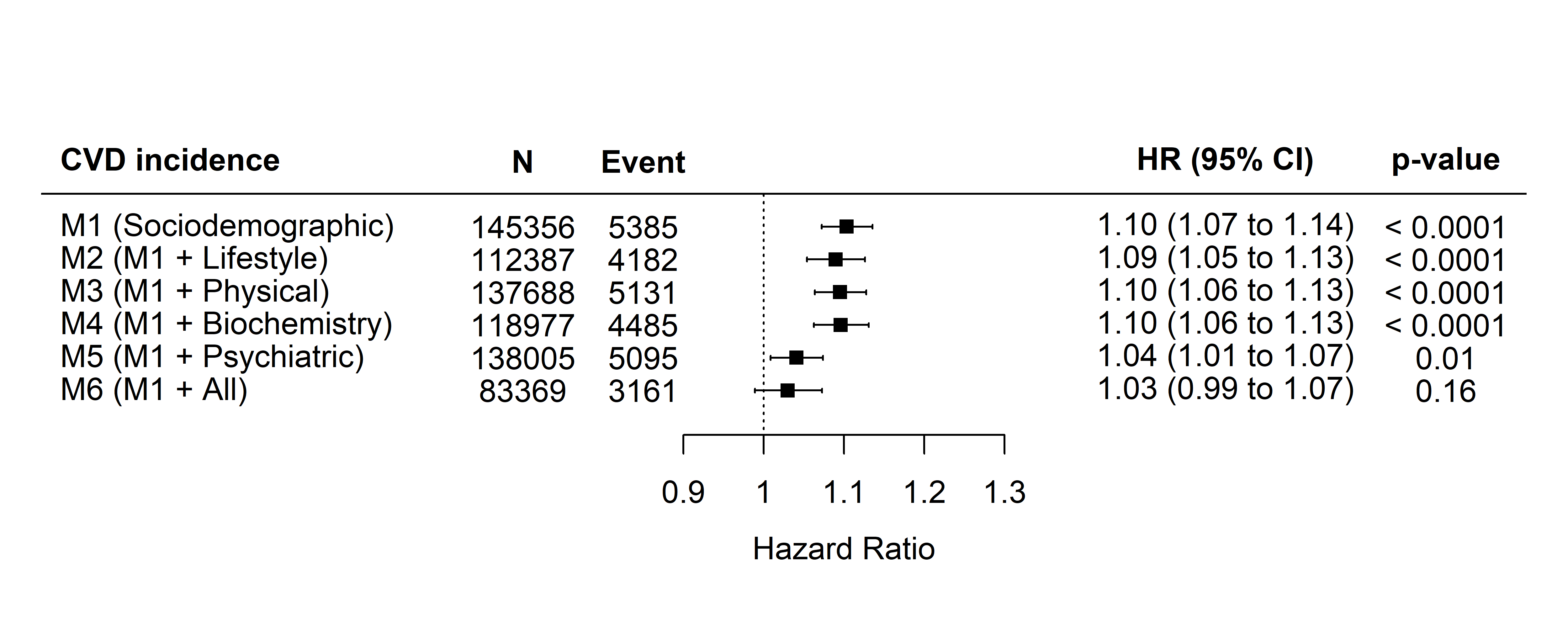


M1: adjusted for age, sex, ethnicity, area-based deprivation index, and education level;
M2: M1 + lifestyle factors: sleep duration, smoking, alcohol drinking, physical activity, TV viewing;
M3: M1 + physical measurements: BMI categories, abdominal obesity, systolic BP, hand grip strength;
M4: M1 + biomarkers: HDL and LDL chlolesterols, glycated haemoglobin, cystatin C, gamma-glutamyltransferase
M5: M1 + psychiatric/emotional factors: diagnoses of depression, anxiety, and schizophrenia, depressive symptoms, anxiety symptoms, any psychosis experience, any behavioural addiction, any drug addition, any self-harm behaviours;
M6: M1 + all potential mediators

Table S1. Characteristics of UK Biobank participants by inclusion in this study

|  | **No (n=350,466)** | **Yes (n=152,040)** |
| --- | --- | --- |
| Mean (SD) age, years | 56.79 (8.23) | 55.92 (7.73) |
| Female | 187710 (53.6) | 85663 (56.3) |
| Ethnicity |  |  |
| White | 324917 (93.5) | 147764 (97.2) |
| Mixed | 2170 ( 0.6) | 788 ( 0.5) |
| South Asian | 8631 ( 2.5) | 1251 ( 0.8) |
| Black | 7001 ( 2.0) | 1060 ( 0.7) |
| Chinese | 1226 ( 0.4) | 348 ( 0.2) |
| Others | 3729 ( 1.1) | 829 ( 0.5) |
| **Socioeconomic status** |  |  |
| Deprivation index | -1.11 (3.19) | -1.72 (2.83) |
| Education level |  |  |
| College or University degree | 91734 (26.5) | 69428 (45.7) |
| A levels/AS levels or equivalent | 34751 (10.0) | 20571 (13.5) |
| O levels/GCSEs or equivalent | 75040 (21.7) | 30150 (19.8) |
| SEs or equivalent | 21266 ( 6.1) | 5619 ( 3.7) |
| NVQ or HND or HNC or equivalent | 25045 ( 7.2) | 7681 ( 5.1) |
| Other professional qualifications | 18100 ( 5.2) | 7703 ( 5.1) |
| None of the above | 74845 (21.6) | 10425 ( 6.9) |
| Prefer not to answer | 5029 ( 1.5) | 463 ( 0.3) |
| **Lifestyle factors** |  |  |
| Sleep duration |  |  |
| <6 hours | 21918 ( 6.3) | 5674 ( 3.7) |
| 6-9 hours | 317016 (91.5) | 144423 (95.2) |
| >9 hours | 7629 ( 2.2) | 1615 ( 1.1) |
| Smoking status |  |  |
| Never | 186125 (53.5) | 87396 (57.6) |
| Former | 119610 (34.4) | 53448 (35.2) |
| Current | 42061 (12.1) | 10917 ( 7.2) |
| Alcohol drinking >14 units / week | 127773 (40.3) | 59454 (42.3) |
| Mean (SD) MET·min of physical activity / week | 2760.64 (2604.90) | 2445.86 (2276.63) |
| Mean (SD) hours of TV viewing / day | 2.95 (1.66) | 2.47 (1.42) |
| **Physical measurements** |  |  |
| BMI categories |  |  |
| Underweight | 1781 ( 0.5) | 848 ( 0.6) |
| Normal | 104361 (30.0) | 58074 (38.3) |
| Overweight | 149329 (42.9) | 62856 (41.4) |
| Obese | 92382 (26.6) | 29939 (19.7) |
| Abdominal obesity | 180623 (51.8) | 65139 (42.9) |
| Mean (SD) systolic blood pressure, mmHg | 138.54 (18.90) | 136.19 (18.07) |
| Mean (SD) grip strength, kg | 30.33 (11.16) | 31.23 (10.70) |
| **Psychiatric/emotional factors** |  |  |
| Depression diagnosed | 20837 ( 5.9) | 7631 ( 5.0) |
| Anxiety diagnosed | 6601 ( 1.9) | 2423 ( 1.6) |
| Schizophrenia diagnosed | 1602 ( 0.5) | 393 ( 0.3) |
| Mean (SD) depressive symptoms (PHQ-9) | 3.66 (4.61) | 2.74 (3.66) |
| Mean (SD) anxiety symptoms (GAD-7) | 2.90 (4.14) | 2.13 (3.38) |
| Any psychotic experience | 361 ( 7.1) | 6942 ( 4.6) |
| Any behavioural addiction | 91 ( 1.8) | 2152 ( 1.4) |
| Any drug addiction | 86 ( 1.7) | 1871 ( 1.2) |
| Any self-harm behaviours | 335 ( 6.4) | 6535 ( 4.3) |
| **Mean (SD) biomarkers** |  |  |
| HDL cholesterol | 1.43 (0.38) | 1.49 (0.38) |
| LDL cholesterol | 3.55 (0.88) | 3.58 (0.84) |
| Glycated haemoglobin | 36.47 (7.27) | 35.32 (5.57) |
| Cystatin C | 0.92 (0.19) | 0.88 (0.15) |
| Gamma-glutamyltransferase | 39.14 (47.86) | 34.00 (35.25) |

Table S2. Participants’ responses to the Childhood Trauma Screener – 5 items (CTS-5)

|  | **Never true** | **Rarely true** | **Sometimes true** | **Often true** | **Very often true** |
| --- | --- | --- | --- | --- | --- |
| **Physical abuse**: People in my family hit me so hard that it left me with bruises or marks | 123533 (81.3) | 16308 (10.7) | **10023 ( 6.6)** | **1321 ( 0.9)** | **855 ( 0.6)** |
| **Emotional abuse**: I felt that someone in my family hated me | 128632 (84.6) | 9219 ( 6.1) | **9937 ( 6.5)** | **2324 ( 1.5)** | **1928 ( 1.3)** |
| **Sexual abuse**: Someone molested me (sexually) | 138700 (91.2) | **7048 ( 4.6)** | **4876 ( 3.2)** | **771 ( 0.5)** | **645 ( 0.4)** |
| **Physical neglect**: There was someone to take me to the doctor if I needed it | **3260 ( 2.1)** | **1212 ( 0.8)** | **4062 ( 2.7)** | 16082 (10.6) | 127424 (83.8) |
| **Emotional neglect**: I felt loved | **2136 ( 1.4)** | **6941 ( 4.6)** | **24545 (16.1)** | 38687 (25.4) | 79731 (52.4) |

Numbers presented are n (%). Red fonts indicate the response that are categorised as child maltreatment

Table S3. Prevalence of child maltreatment in UK Biobank

|  | **Overall (n=152,040)** | **Female (n=85,663)** | **Male (n=66,377)** | **Age 38-50 (n=40,861)** | **Age 51-60 (n=60,318)** | **Age 61-72 (n=50,861)** |
| --- | --- | --- | --- | --- | --- | --- |
| Any child maltreatment | 50602 (33.3) | 29725 (34.7) | 20877 (31.5) | 14717 (36.0) | 20306 (33.7) | 15579 (30.6) |
| *By number of maltreatment* |  |  |  |  |  |  |
| 0 | 101438 (66.7) | 55938 (65.3) | 45500 (68.5) | 26144 (64.0) | 40012 (66.3) | 35282 (69.4) |
| 1 | 30915 (20.3) | 17134 (20.0) | 13781 (20.8) | 8407 (20.6) | 12436 (20.6) | 10072 (19.8) |
| 2 | 11625 ( 7.6) | 7082 ( 8.3) | 4543 ( 6.8) | 3515 ( 8.6) | 4582 ( 7.6) | 3528 ( 6.9) |
| 3 | 5219 ( 3.4) | 3457 ( 4.0) | 1762 ( 2.7) | 1797 ( 4.4) | 2110 ( 3.5) | 1312 ( 2.6) |
| 4 | 2153 ( 1.4) | 1499 ( 1.7) | 654 ( 1.0) | 739 ( 1.8) | 895 ( 1.5) | 519 ( 1.0) |
| 5 | 690 ( 0.5) | 553 ( 0.6) | 137 ( 0.2) | 259 ( 0.6) | 283 ( 0.5) | 148 ( 0.3) |
| *By types of maltreatment* |  |  |  |  |  |  |
| Physical abuse | 12199 (8.02) | 6745 (7.87) | 5454 (8.22) | 4163 (10.19) | 4975 (8.25) | 3061 (6.02) |
| Emotional abuse | 14189 (9.33) | 9666 (11.28) | 4523 (6.81) | 5103 (12.49) | 5677 (9.41) | 3409 (6.70) |
| Sexual abuse | 13340 (8.77) | 9462 (11.05) | 3878 (5.84) | 4001 (9.79) | 5482 (9.09) | 3857 (7.58) |
| Physical neglect | 8534 (5.61) | 5108 (5.96) | 3426 (5.16) | 2074 (5.08) | 3215 (5.33) | 3245 (6.38) |
| Emotional neglect | 33622 (22.11) | 19449 (22.70) | 14173 (21.35) | 9738 (23.83) | 13576 (22.51) | 10308 (20.27) |

Numbers presented are n (%)

Table S4. Association of potential mediators and CVD

|  | **HR (95% CI)** | **P** |
| --- | --- | --- |
| **Lifestyle factors** |  |  |
| Sleep duration |  |  |
| 6-9 hours | 1 (Reference) |  |
| >9 hours | 1.08 (0.83–1.41) | 0.57 |
| <6 hours | 1.34 (1.16–1.54) | < 0.0001 |
| Smoking status |  |  |
| Never | 1 (Reference) |  |
| Former | 1.12 (1.05–1.20) | 0.001 |
| Current | 1.18 (1.05–1.33) | 0.007 |
| Alcohol drinking >14 units/week | 1.04 (0.97–1.12) | 0.23 |
| MET·min of physical activity/week | 1.00 (1.00–1.00) | 0.34 |
| Hours of TV viewing/day | 1.03 (1.01–1.05) | 0.008 |
| **Physical measurements** |  |  |
| BMI categories |  |  |
| Underweight | 1.06 (0.60–1.87) | 0.85 |
| Normal | 1 (Reference) |  |
| Overweight | 1.09 (1.00–1.18) | 0.049 |
| Obese | 1.22 (1.10–1.35) | 0.0001 |
| Abdominal obesity | 1.01 (0.94–1.09) | 0.78 |
| Systolic blood pressure, mmHg | 1.01 (1.00–1.01) | < 0.0001 |
| Grip strength, kg | 1.00 (0.99–1.00) | 0.03 |
| **Psychiatric/emotional factors** |  |  |
| Depression diagnosed | 0.89 (0.76–1.05) | 0.17 |
| Anxiety diagnosed | 1.08 (0.85–1.39) | 0.52 |
| Schizophrenia diagnosed | 0.70 (0.33–1.48) | 0.35 |
| Depressive symptoms (PHQ-9) | 1.05 (1.04–1.06) | < 0.0001 |
| Anxiety symptoms (GAD-7) | 0.98 (0.96–1.01) | 0.07 |
| Any psychotic experience | 1.02 (0.88–1.19) | 0.76 |
| Any behavioural addiction | 1.06 (0.83–1.37) | 0.63 |
| Any drug addiction | 1.26 (0.97–1.63) | 0.08 |
| Any self-harm behaviours | 1.07 (0.90–1.28) | 0.42 |
| **Biomarkers** |  |  |
| HDL cholesterol | 0.67 (0.60–0.75) | < 0.0001 |
| LDL cholesterol | 0.95 (0.89–1.01) | 0.06 |
| Glycated haemoglobin | 1.02 (1.01–1.02) | < 0.0001 |
| Cystatin C | 1.76 (1.52–2.03) | < 0.0001 |
| Gamma-glutamyltransferase | 1.00 (1.00–1.00) | 0.007 |

All factors listed were mutually adjusted, and were additional adjusted for number of maltreatment, age, sex, ethnicity, area-based deprivation index, and education level.

Table S5. Mediation analysis of number of child maltreatment and CVD using sex-specific biomarker z-scores

|  | **Outcome model ^a^** | | **Mediator model ^b^** | | **Percent mediated** |  |
| --- | --- | --- | --- | --- | --- | --- |
|  | **HR (95% CI)** | **P** | **OR / β (95% CI)** | **P** |  | **P** |
| Short sleeper | 1.24 ( 1.10, 1.41) | 0.0007 | 1.14 ( 1.11, 1.18) | < 0.0001 | 2.4 | 0.04 |
| Smoker/ex-smoker | 1.13 ( 1.07, 1.20) | < 0.0001 | 1.20 ( 1.18, 1.21) | < 0.0001 | 14.5 | 0.02 |
| Obesity | 1.14 ( 1.07, 1.22) | < 0.0001 | 1.05 ( 1.03, 1.06) | < 0.0001 | 1.9 | 0.06 |
| TV viewing time | 1.03 ( 1.00, 1.06) | 0.03 | 0.00 (-0.01, 0.00) | 0.13 | - | - |
| Systolic BP | 1.12 ( 1.09, 1.15) | < 0.0001 | -0.02 (-0.02, -0.01) | < 0.0001 | - | - |
| Handgrip strength | 0.96 ( 0.93, 1.00) | 0.07 | 0.00 (-0.01, 0.00) | 0.68 | - | - |
| Depressive symptom | 1.18 ( 1.15, 1.21) | < 0.0001 | 0.22 ( 0.22, 0.23) | < 0.0001 | 55.7 | < 0.0001 |
| HDL Cholesterol | 0.87 ( 0.85, 0.90) | < 0.0001 | -0.02 (-0.03, -0.01) | < 0.0001 | 8.2 | 0.03 |
| Glycated haemoglobin | 1.11 ( 1.09, 1.13) | < 0.0001 | 0.00 ( 0.00, 0.01) | 0.38 | - | - |
| Cystatin C | 1.09 ( 1.07, 1.11) | < 0.0001 | 0.00 ( 0.00, 0.01) | 0.12 | - | - |
| Gamma-glutamyltransferase | 1.05 ( 1.03, 1.07) | < 0.0001 | 0.00 ( 0.00, 0.01) | 0.22 | - | - |

^a^ Outcome model: CVD regressed by potential mediators;
^b^ Mediator model: potential mediator regressed by child maltreatment.
ORs were presented for short sleeper, smoker/ex-smoker, and obesity

Table S6. Mediation analysis of number of child maltreatment and CVD excluding biomarkers

|  | **Outcome model ^a^** | | **Mediator model ^b^** | | **Percent mediated** |  |
| --- | --- | --- | --- | --- | --- | --- |
|  | **HR (95% CI)** | **P** | **OR / β (95% CI)** | **P** |  | **P** |
| Short sleeper | 1.24 ( 1.10, 1.41) | 0.0007 | 1.14 ( 1.11, 1.18) | < 0.0001 | 2.6 | 0.01 |
| Smoker/ex-smoker | 1.13 ( 1.07, 1.20) | < 0.0001 | 1.20 ( 1.18, 1.21) | < 0.0001 | 14.1 | 0.01 |
| Obesity | 1.14 ( 1.07, 1.22) | < 0.0001 | 1.05 ( 1.03, 1.06) | < 0.0001 | 7.3 | 0.02 |
| TV viewing time | 1.03 ( 1.00, 1.06) | 0.03 | 0.00 (-0.01, 0.00) | 0.13 | - | - |
| Systolic BP | 1.12 ( 1.09, 1.15) | < 0.0001 | -0.02 (-0.02, -0.01) | < 0.0001 | - | - |
| Handgrip strength | 0.96 ( 0.93, 1.00) | 0.07 | 0.00 (-0.01, 0.00) | 0.68 | - | - |
| Depressive symptom | 1.18 ( 1.15, 1.21) | < 0.0001 | 0.22 ( 0.22, 0.23) | < 0.0001 | 57.6 | < 0.0001 |

^a^ Outcome model: CVD regressed by potential mediators;
^b^ Mediator model: potential mediator regressed by number of ACEs.
ORs were presented for short sleeper, smoker/ex-smoker, and obesity
